# The UK SPIRIT 1 trial in newly-diagnosed chronic myeloid leukaemia (CML)

**DOI:** 10.1111/bjh.17961

**Published:** 2022-01-06

**Authors:** Paolo Gallipoli, Richard E Clark, Jenny Byrne, Jane F Apperley, Dragana Milojkovic, Letizia Foroni, John M Goldman, Stephen O’Brien

**Affiliations:** 1 Centre for Haemato-Oncology, Barts Cancer Institute, Queen Mary University of London, London, UK; 2 Department of Molecular & Clinical Cancer Medicine, University of Liverpool, Liverpool, UK; 3 Department of Haematology, Nottingham University Hospital Trust, Nottingham, UK; 4 Centre for Haematology, Imperial College London at Hammersmith Hospital, London, UK; 5 Faculty of Medical Sciences, Newcastle University, Newcastle, UK

**Keywords:** Chronic Myeloid Leukaemia, Interferon, Tolerability, side-effects, molecular responses, Clinical trials

Although the UK National Cancer Research Institute’s SPIRIT 1 trial closed in 2009 due to poor recruitment, we thought it would be useful to the haematology community to report the outcome. This study closed early as it failed to recruit adequate numbers and our present report addresses the reasons, makes some observations about tolerability and the difficulty of delivering interferon-alpha (IFN) in the UK. The SPIRIT 1 study (EUDRACT Number 2004-001622-24) was originally conceived in conjunction with other international groups, and patients with newly-diagnosed chronic phase CML were randomised to receive either standard or higher-dose imatinib or imatinib in combination with PEGylated IFN (PEG-IFN).

There have been four sizeable studies comparing imatinib with imatinib combined with IFN. In the French study^1^, although complete cytogenetic response rates were similar at 12 months between the arms, a higher rate of deep molecular response (reduction in BCR-ABL transcripts measured by qPCR) was seen in the imatinib + PEG-IFN group. The Nordic study^2^ also showed a superior molecular response rate for imatinib + PEG-IFN combination therapy. In contrast, neither the German study^3^ nor a study from the MD Anderson Cancer Centre^4^ showed a significant difference in molecular response rates. None of these combination studies have demonstrated a superior progression-free (PFS) or overall survival (OS) advantage for patients who received combination treatment.

In this context, between June 2005 and January 2009, 258 patients in the UK with newly-diagnosed CML were randomised 1:1:1 to one of 3 treatment groups: 1) imatinib 400mg daily: 2) imatinib 800mg daily or; 3) imatinib 400mg daily plus PEG-IFN at a starting dose of 90μg per week, escalating to 180μg per week if tolerated. The primary endpoint for the SPIRIT 1 study was 5 year OS. The study was powered to show an improvement of 6% in OS and the predicted sample size was 822 patients per arm, 2,466 in total. By 2008 it was evident that recruitment was slow and we undertook a survey of clinicians to help us understand the reasons. Investigators gave a clear message that PEG-IFN was increasingly unpopular with patients and physicians due to side effects and inconvenience of administration. As a result, the imatinib/PEG-IFN combination arm was closed. The advent of newer tyrosine kinase inhibitors (TKIs) rendered the trial obsolete and it was terminated early, having recruited 10.5% (258/2,466) of the required number of patients.

Tables I & II show the characteristics, outcomes and toxicities observed in the study. As the study was underpowered, formal statistical analyses would be unreliable but no striking outcome differences are evident. The main limiting toxicities in the imatinib/PEG-IFN arm were grade 3/4 neutropenia, low-grade fatigue/flu-like symptoms and a small but not inconspicuous incidence of mood changes, mainly depression, with some being grade 3/4. These might have all contributed to the difficulty in delivering the combination treatment and the lack of popularity of PEG-IFN amongst both clinicians and patients. Adverse events in the other 2 arms were as previously reported, with high dose imatinib showing higher rates of limiting thrombocytopenia and low-grade gastrointestinal, musculoskeletal and skin-related adverse events compared to the other 2 arms.

The closure of SPIRIT 1 essentially brought to an end the use of PEG-IFN for the treatment of CML in the UK apart from some special circumstances such as pregnancy. Some of the aforementioned studies have had similar levels of adverse events which led, for example, to 45% of patients discontinuing PEG-IFN in the first year of the French study^1^. However a few, mainly European, countries continue to advocate the use of PEG-IFN in combination with imatinib and other TKIs^5–7^ and it is possible that, given a mechanism of action that is different to that of TKIs although not well understood, PEG-IFN may have a role to play in the management of CML^8^. There is the suggestion that IFN may offer the benefit of higher rates of treatment-free remission than with a TKI alone^9^ but these findings have not been validated.

Imatinib is such a clinically and cost-effective treatment for the majority of patients with CML^10^ that it is probably impossible to demonstrate any significant additional survival benefit from adding PEG-IFN and the burden of more toxicity, inconvenience and cost has led to its abandonment, rightly or wrongly, for the routine management of CML in the UK. Imatinib 400mg daily remains the predominant first line therapy in this country.

## Figures and Tables

**Table I:** Patient characteristics and main outcomes. Statistical comparisons have not been performed as the number of patients recruited was insufficient to allow meaningful analysis. Key: MR3 = BCR-ABL/ABL ratio of <0.1; MR4 = BCR-ABL/ABL ratio of <0.01; MR4.5 = BCR-ABL/ABL ratio of <0.0032,;MCyR = major cytogenetic response; MMR = major molecular response; CCyR = complete cytogenetic response; CHR = complete haematological response.

		Imatinib 400mg n=98 (%)	Imatinib 800mg n=96 (%)	Imatinib + PEG-IFN n=64 (%)	Total N=258 (%)
**Mean age**		52.5, range 18-79	52.2, range 19-80	53.8, range 31-76	52.7, range 18-80
**Gender distribution: male/female %**		61.2 / 38.8	62.5 / 37.5	65.6 / 34.4	62.8 / 37.2
**Overall survival: alive at 5 years (%)**		88 (89.8)	90 (93.8)	55 (85.9)	233 (90.3)
**Molecular response at 1 year**	**MR3**	23 (23.5)	33 (34.4)	18 (28.1)	74 (28.7)
	**MR4**	7 (7.1)	9 (9.4)	10 (15.6)	26 (10.1)
	**MR4.5**	3 (3.1)	3 (3.1)	5 (7.8)	11 (4.3)
**Time to progression (months) Median, range**		58.46, 0.2-92.1	33.46, 0.7-79.1	12.55, 0.3-61.6	29.90, 0.2-92.1
**Time to Treatment Failure (months) Median, range**		58.87, 0.2-93.2	59.43, 4.8-102.2	55.75, 0.3-95.8	58.94, 0.2-102.2
**CHR at any time**		93 (94.9)	88 (91.7)	61 (95.3)	242 (93.8)
**MCyR at 1 year**		35 (35.7)	36 (37.5)	16 (25)	87
**CCyR at 1 year**		26 (26.5)	31 (32.3)	14 (21.9)	71

**Table II:** Main adverse events

	Imatinib 400mg (98)				Imatinib 800mg (96)				Imatinib 400mg + PEG-Interferon (64)			
	All Grades		Grade 3-4		All Grades		Grade 3-4		All Grades		Grade 3-4	
Events	n	%	n	%	n	%	n	%	n	%	n	%
**Neutropenia**	12	12.2	10	10.2	15	15.6	10	10.4	24	37.5	21	32.8
**Anaemia**	7	7.1	2	2	12	12.5	0	0	8	12.5	3	4.6
**Thrombocytopenia**	7	7.1	3	3	24	25	13	13.5	9	14	5	7.8
**Nausea/vomiting**	29	29.6	1	1	42	43.8	3	3.1	17	26.6	0	0
**Abdominal pain**	13	13.3	1	1	16	16.7	1	1	12	18.8	2	3.1
**Diarrhoea**	26	26.5	2	2	37	38.5	6	6.3	16	25.0	1	1.6
**Fatigue**	20	20.4	0	0	29	30.2	2	2.1	26	40.6	1	1.6
**Flu-like symptoms**	5	5.1	0	0	4	4.2	1	1	13	20.3	1	1.6
**Infection**	39	39.8	5	5.1	29	30.2	2	2.1	22	34.4	5	7.8
**Muscle/bone pain**	50	51.0	5	5.1	62	64.6	6	6.3	34	53.1	3	4.7
**Skin rash**	35	35.7	4	4.1	43	44.8	6	6.3	23	35.9	7	10.9
**Depression/mood changes**	7	7.1	0	0	9	9.3	0	0	11	17.2	3	4.7
